# Stage-Specific Transcriptomes of the Mussel *Mytilus coruscus* Reveals the Developmental Program for the Planktonic to Benthic Transition

**DOI:** 10.3390/genes14020287

**Published:** 2023-01-21

**Authors:** Yu-Qing Wang, Qi Liu, Yan Zhou, Lizhi Chen, Yue-Ming Yang, Xue Shi, Deborah M. Power, Yi-Feng Li

**Affiliations:** 1 International Research Center for Marine Biosciences, Ministry of Science and Technology, Shanghai Ocean University, Shanghai 201306, China; 2 Key Laboratory of Exploration and Utilization of Aquatic Genetic Resources, Ministry of Education, Shanghai Ocean University, Shanghai 201306, China; 3 Aquatic Technology Promotion Station, Sanmen Rural Bureau, Taizhou 317199, China; 4 Comparative Endocrinology and Integrative Biology, Centro de Ciências do Mar (CCMAR), Universidade do Algarve, Campus de Gambelas, 8005-139 Faro, Portugal

**Keywords:** *Mytilus coruscus*, transcriptome, larval settlement and metamorphosis, pediveliger larvae, hard-shelled mussel

## Abstract

Many marine invertebrate larvae undergo complex morphological and physiological changes during the planktonic—benthic transition (a.k.a. metamorphosis). In this study, transcriptome analysis of different developmental stages was used to uncover the molecular mechanisms underpinning larval settlement and metamorphosis of the mussel, *Mytilus coruscus*. Analysis of highly upregulated differentially expressed genes (DEGs) at the pediveliger stage revealed enrichment of immune-related genes. The results may indicate that larvae co-opt molecules of the immune system to sense and respond to external chemical cues and neuroendocrine signaling pathways forecast and trigger the response. The upregulation of adhesive protein genes linked to byssal thread secretion indicates the anchoring capacity required for larval settlement arises prior to metamorphosis. The results of gene expression support a role for the immune and neuroendocrine systems in mussel metamorphosis and provide the basis for future studies to disentangle gene networks and the biology of this important lifecycle transformation.

## 1. Introduction

Mussels are important aquaculture species worldwide, and several different species are produced along the coast of China. The aquaculture mussel production in China was about 829,481 tons in 2021 [1]. One important factor in the productivity of the mussel aquaculture industry is larval recruitment, and settlement and metamorphosis are important processes that determine its success. Since the early life stages have very high mortality rates in bivalves, a better understanding of these stages is vital for aquaculture and restoration ecology [2]. In addition to their importance for aquaculture, mussels, the same as other bivalves, provide important ecosystem services in coastal marine food webs by providing habitats, food, and benthic–pelagic coupling through filtering organic detritus and microalgae [3].

The same as many other marine invertebrates, the lifecycle of mussels can be divided into two-phases, a planktonic larval phase and a sessile sedentary adult phase. The free-swimming larvae (larval form) transform into a sessile juvenile through a process known as metamorphosis [4]. Metamorphosis occurs across the animal kingdom and involves a shift in body form and physiology and the loss of larval traits and the acquisition of adult traits. Mussel larvae acquire “metamorphic competence” at the pediveliger stage when larvae settle and the metamorphic transition initiates [5,6]. Mussel settlement is a behavioral response, and when larvae encounter an appropriate substrate, they anchor themselves to it by producing byssal threads [7].

The irreversible morphological and physiological changes associated with the development of juvenile-specific structures during metamorphosis include the loss of the velum, development of the gills, and secretion of the juvenile shell [8]. The complex process of settlement that precedes and is necessary for metamorphosis requires that the larvae can sense their environment through, for example, chemical cues, and then relay the signal through neuroendocrine or neuroendocrine-immune signaling pathways to trigger metamorphosis [6,7,9].

The factors that determine bivalve settlement and metamorphosis are still enigmatic. In the oyster, *Crassostrea virginica*, a study from the early 1990s, reported the dopaminergic pathway governs settlement and the adrenergic pathway metamorphosis [9]. Subsequent studies of the adrenergic receptor genes corroborated the importance of this system in the metamorphosis of the hard-shelled mussel (*M. coruscus*) and oyster (*Crassostrea angulata*) [10,11]. Furthermore, the metamorphosis of many marine invertebrates, such as the bryozoan (*Bugula neritina*), mussel (*M. coruscus*), and oyster (*C. gigas*), is negatively regulated by the nitric oxide (NO) signaling pathway [12,13,14].

Recently, transcriptome studies have started to transform the understanding of the larval to juvenile transition of marine invertebrate species [15,16,17]. For example, a microarray study of the abalone (*Haliotis asinina*) identified 144 genes involved in the acquisition of larval “competence” and metamorphosis [17]. The transcriptome analysis gives an overview of genes with changing expression profiles during larval settlement and metamorphosis and allows the identification of candidates’ regulatory factors. A better understanding of metamorphosis would contribute to improve larval survival during hatchery production [12]. The present study was designed to advance our understanding and provide the basis for future investigations of the molecular and organismal changes occurring during metamorphosis in the hard-shelled mussels (*M. coruscus*). For this reason, transcriptomes were developed for unambiguous developmental stages that can be easily identified and collected. The global changes in gene expression between developmental stages were used to uncover the candidate genes and pathways that underpin the profound organismal and physiological changes during metamorphosis.

## 2. Materials and Methods

### 2.1. Sample Acquisition

Spawning and larvae culture techniques of the hard-shelled mussels (*M. coruscus*) were performed as previously described [4]. Briefly, adult mussels (~120) were cleaned and kept in 10 L tanks containing aerated seawater (salinity: 30 ppt; pH: 8.1) after arrival in the laboratory. Mussels were induced to spawn by removing them from seawater and exposing them to air overnight at room temperature. Mussels were transferred back to the 10 L tanks containing aerated, filtered seawater (FSW; acetate–fiber filter: 1.2 μm pore size) at 18 °C. When mussels started spawning, they were gently transferred to individual 2 L glass beakers, and eggs and sperm were collected by filtration. Fertilization was performed by gently mixing equal volumes of sperm and eggs in FSW at 18 °C. Excess sperm was removed after egg fertilization by filtering the mixture through a nylon plankton net (mesh size: 20 μm), and the fertilized eggs were collected and kept in a 2 L glass beaker containing FSW for two days at 18 °C. Larvae were maintained at a density of 5 larvae mL^−1^ FSW at 18 °C by feeding with the microalgae *Isochrysis zhanjiangensis* (50,000 cells ml^−1^). The following developmental stages were collected: (1) trochophore larvae (1 day post-fertilization, dpf), (2) D-veliger larvae (4 dpf), (3) umbo larvae (15 dpf), (4) pediveliger larvae (41 dpf), and (5) juvenile (post-metamorphic, 63 dpf). Samples of the developing larvae were collected by filtering the FSW through a nylon plankton net (mesh size: 40 μm) and rinsing with sterile FSW. The small size of the larvae and juveniles meant that samples for analysis consisted of pools of larvae (containing approximately 300–3000 individuals/sample) that were collected into microcentrifuge tubes and snap-frozen immediately by immersion in liquid nitrogen. For each developmental stage, 5 samples were collected for RNA extraction and sequencing.

All procedures for mussel acclimation and experimentation were authorized by the Animal Ethics Committee of Shanghai Ocean University with the registration number SHOU-DW-2018-013.

### 2.2. RNA Extraction, cDNA Library Construction, and Illumina Sequencing

Total RNA was extracted from samples of each developmental stage using RNAiso Plus reagent according to the manufacturer’s instructions (Takara, Japan). Contaminating genomic DNA was eliminated using DNase (Ambion Turbo DNase kit). The quality of the DNase-treated total RNA samples was determined using a 2100 Bioanalyzer (Agilent Technologies, Inc., Santa Clara, CA, USA) and quantified using a NanoDrop (ND-2000, Thermo Scientific, Wilmington, DE, USA). Only high-quality RNA samples (RIN ≥ 8.0) were used to construct the sequencing library. Four biological replicates were used to generate a sequencing library for each developmental stage. Twenty sequencing libraries were generated using an Illumina TruSeq^TM^ RNA sample preparation kit (San Diego, CA, USA). RNA-seq libraries were sequenced using an Illumina Hiseq xten/NovaSeq 6000 sequencer (Illumina, San Diego, CA, USA) for 2 × 150 bp paired-end reads at Majorbio Bio-Pharm Biotechnology Co., Ltd. (Shanghai, China). Raw sequence data were submitted to the NCBI Sequence Read Archive (SRA) database under accession number SRP300586.

### 2.3. Transcriptome Analysis

Clean reads were mapped to the *M. coruscus* genome (https://www.ncbi.nlm.nih.gov/assembly/GCA_017311375.1) (accessed on 10 March 2021) [18] using HISAT v2.0.4 with default values, and the reads from each sample were mapped separately [19]. RSEM (RNA-Seq by Expectation Maximization) was used to analyze the expression level of each gene transcript using the transcript per million mapped reads (TPM) method [20]. Differently expressed genes (DEGs) were identified using DESeq2 [21] in R software (v. 1.24.0., Mark D. Robinson, Darlinghurst, NSW, AUS, http://bioconductor.org) (accessed on 10 March 2021). DEGs between different developmental stages were screened by using FDR ≤ 0.05. KOBAS 2.1.1 (http://kobas.cbi.pku.edu.cn/home.do) (accessed on 10 March 2021) was used to identify DEGs in KEGG pathways using |log2FC| ≥ 1 as the cut-off according to Fisher’s exact test, with *p*-values adjusted through Benjamini–Hochberg multiple testing correction [22]. Annotation of the unigenes was established using BLASTX to interrogate Nr (NCBI non-redundant protein sequences), Nt (NCBI non-redundant nucleotide sequences), KO (KEGG Ortholog database), GO (Gene Ontology), KOG/COG (Clusters of Orthologous Groups of proteins), Pfam (Protein family), and Swiss-Prot database (a manually annotated and reviewed protein sequence database) based on sequence similarity using an e-value cut-off of 10^−5^. Gene ontology (GO) terms (biological processes, molecular functions, and cellular components) were assigned to the dataset using the BLAST2GO program [23].

### 2.4. qPCR Analysis

DEGs in the pediveliger stage with > 2-fold difference in expression compared to all other stages were selected for corroboration by quantitative real-time PCR (qPCR). Thirteen genes linked to neuroendocrine (n = 4), growth and apoptosis (n = 4), and immune (n = 3) and other highly modified genes (n = 2) were selected for qPCR (Table S1). Primers were designed using the sequences obtained from the Illumina sequencing data (Table S1). Five samples were analyzed per stage and included the four total RNA samples used for sequencing. Total RNA was treated with DNase in a final reaction volume of 10 μL containing 1 μL gDNA Eraser, 2 μL 5 × gDNA Eraser buffer, 500 ng total RNA, and RNase-free dH_2_O at 42 °C for 2 min. Reverse transcription was carried out in a 20 μL reaction volume by adding 4 μL 5× PrimeScript Buffer 2, 1 μL RT Primer Mix, 1 μL PrimeScript RT Enzyme Mix I, and 4 μL RNase Free dH_2_O. The reactions were performed at 37 °C for 15 min and followed by 5 s at 87 °C.

qPCR analysis was performed with duplicate reactions using five biological replicates/stage in 96 multi-well plates using a LightCycler 960 (Roche). The standard curve method was used for absolute quantification. PCR amplicons were sequenced to confirm their identity and used to generate standard curves that ranged from 10^7^–10^1^ cDNA copies in qPCR experiments. qPCR reactions contained 1 μL template cDNA (around 20 ng), 0.3 μL of the forward, 0.3 μL of the reverse primers (10 μM), 5 μL of 2 × FastStart Essential DNA Green Master (Roche), and sterile Milli-Q water to give a final reaction volume of 10 μL. Amplification was carried out with the following protocols: 10 min at 95 °C, followed by 45 cycles of 10 s at 95 °C and 10 s at 57 °C. Melting curve analysis confirmed single reaction products with all the primers used. qPCR data were analyzed using the Wilcoxon/Kruskal–Wallis test using JMP^TM^ software (Version 10.0.0), and *p* < 0.05 was considered statistically significant.

## 3. Results

### 3.1. Transcriptome Analysis

**Figure 1:**
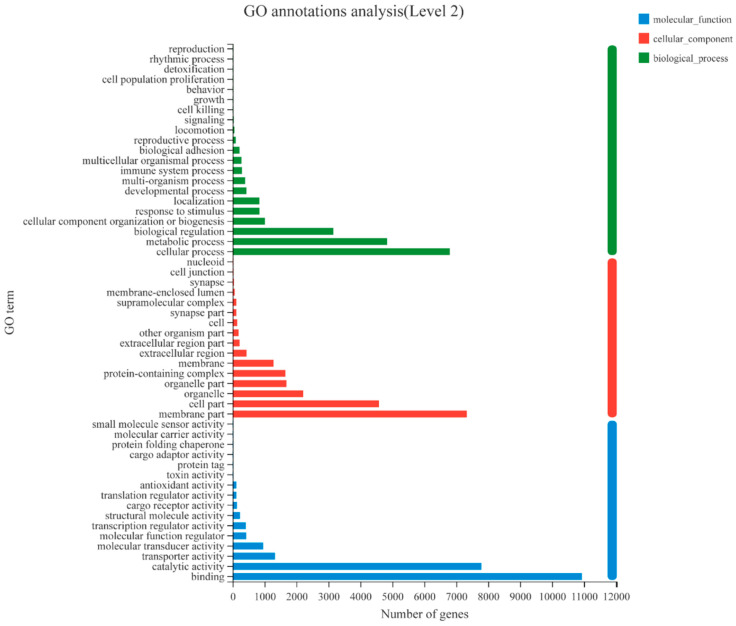
Gene ontology (GO) term distribution of transcripts. The GO annotation results were based on 20,199 transcripts detected at least in one of the five developmental stages. Gene ontology categories included molecular function, cellular component, and biological process. GO categories for each function were sorted by the increasing number of genes enriched in the process.

The mussel transcriptome sequences of different developmental stages yielded a total of 37,478 transcripts annotated by the GO/KEGG/COG/NR/Swiss-Prot/Pfam databases, and 33,576 could be detected in at least one of the five developmental stages (Table S6). The Q30 score for the sequences ranged from 92% to 95%. GO-annotated unigenes were assigned to three major functional categories, with 21 subcategories in biological processes, 16 subcategories in cellular processes, and 16 subcategories in molecular function. Cellular processes (GO: 0009987) were the dominant group in the biological processes category, followed by metabolic processes (GO: 0008152) and biological regulation (GO: 0065007) (Figure 1). Membrane parts (GO: 0044425), cell parts (GO: 0044464), and organelles (GO: 0043226) were the three dominant groups in the cellular components category (Figure 1). The three groups with the highest number of genes enriched in the cellular component category were binding (GO: 0005488), catalytic activity (GO: 0003824), and transporter activity (GO: 0005215) (Figure 1).

### 3.2. Differential Gene Expression between Five Developmental Stages of Mussel

**Figure 2:**
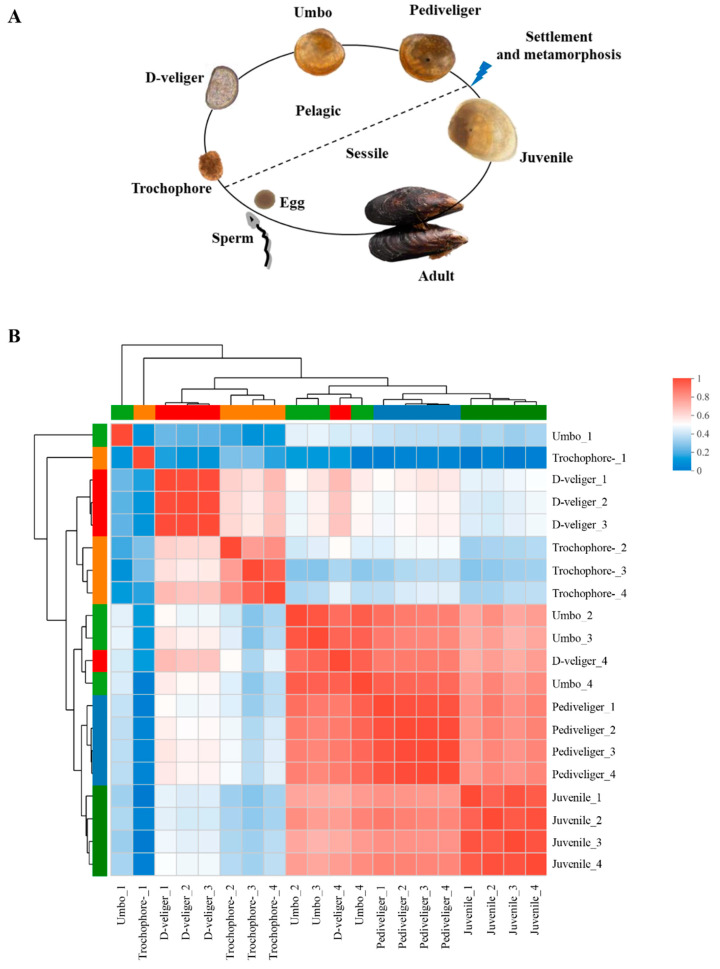
The life cycle of the mussel *M. coruscus* (**A**) and heat map for the Pearson correlation coefficient for all 20 samples (**B**). The heat map plots the correlation coefficient score between any two samples. The color of a square represents each coefficient in the correlation matrix. The color scale used is blue to red, with blue representing 0 and red representing 1. The five clusters defined by hierarchical clustering are indicated at the top.

The life cycle of *M. coruscus* was presented in Figure 2A. From the correlation of gene expression, there is a strong correlation between the trochophore larvae stage and D-veliger larvae stage, as well as umbo larvae stage, pediveliger larvae stage, and juvenile stage. Differences between the D-veliger larvae stage and umbo larvae stage started to emerge, suggesting these two stages may be the key transition period of the metamorphosis and development of *M. coruscus* (Figure 2B).

**Figure 3:**
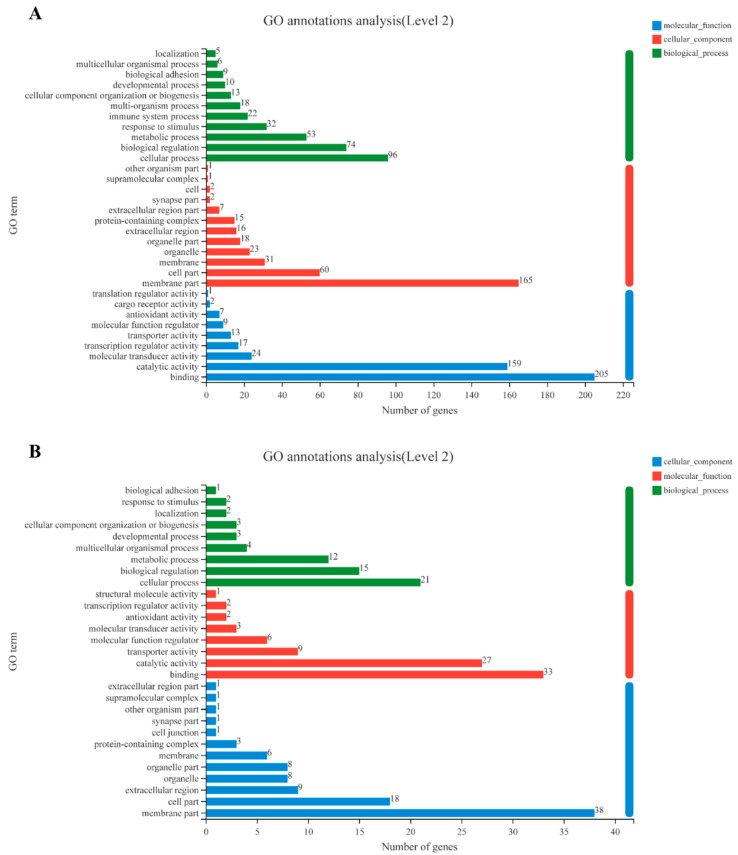
Gene ontology (GO) term enrichment analysis. The GO annotation results were based on the 684 increased DEGs (screened with 2-fold change in the pediveliger stage in comparison with other developmental stages) (**A**) or the 121 DEGs decreased (**B**). Gene ontology categories included molecular function, cellular component, and biological process. GO categories for each function were sorted by increasing order of gene numbers, based on the GO enrichment test; *p*-value < 0.05.

**Figure 4:**
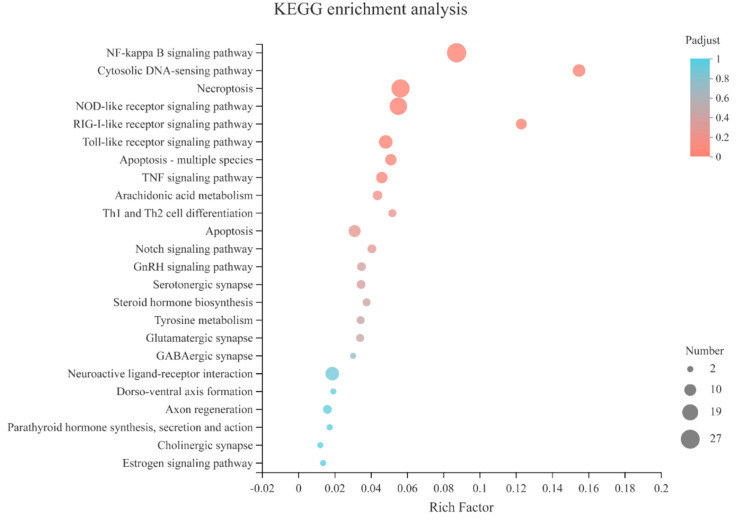
KEGG pathway enrichment scatter plot of the DEGs. The vertical axis represents the path name, and the horizontal axis represents the path factor corresponding to the rich factor. The color of the point represents the size of the *p*−value. The smaller the *p*−value, the closer the color is to red. The number of differential genes included in each pathway is presented by the point’s size.

The pediveliger stage is an important pre-metamorphic stage, since this is when “competence” is acquired and juvenile structures start to develop, and reception of an external stimulus triggers metamorphosis. For this reason, the change in gene transcripts in the pediveliger stage relative to all other stages was explored in detail. DEGs in the pediveliger stage with >two-fold increase (Table S2) or decrease (Table S3) in expression compared to all other stages were selected and GO ontology and KEGG enrichment analysis established (Figure 3 and Figure 4). The number of DEGs with > two-fold increase in expression was higher than DEGs with >two-fold decrease in expression in the biological process, cellular component, and molecular function classification, although their predominant processes were similar in each classification (Figure 3). Among the GO terms enriched by DEGs with increased expression, cellular processes, membrane fraction, and binding were the top three enrichment categories at the biological process, cellular fraction, and molecular function levels, respectively (Figure 3A), and the cellular processes, membrane fraction, and binding remained the top three categories among the GO terms for reduced genes expression (Figure 3B).

The enriched KEGG pathways identified 24 pathways that were significantly overrepresented in the DEGs of the pediveliger stage with >two-fold increase in expression compared to all other stages (Figure 4, Table S4). The identification of pathways related to the endocrine system (GnRH secretion, parathyroid hormone synthesis, secretion and action, and estrogen signaling pathway) and the nervous system (Serotonergic synapse, glutamatergic synapse, GABAergic synapse, neuroactive ligand–receptor interaction, and cholinergic synapse), substantiated the data quality as it corroborated previously published works [4,24,25,26,27,28,29]. Further, significantly overrepresented KEGG pathways were linked to the immune system (NF-kappa B signaling pathway, cytosolic DNA-sensing pathway, NOD-like receptor signaling pathway, RIG-I-like receptor signaling pathway, Toll-like receptor signaling pathway, TNF-signaling pathway, and Th1 and Th2 cell differentiation) (Figure 4, Table S4). The enriched pathways of “necroptosis”, “apoptosis”, “Notch signaling pathway”, “dorsoventral axis formation”, and “axon regeneration” are coherent with the loss of larval structures in the pediveliger stage and the acquisition of juvenile structures (Figure 4, Table S4).

### 3.3. qPCR Analysis of Differential Gene Expression during Development of Mussel

**Figure 5:**
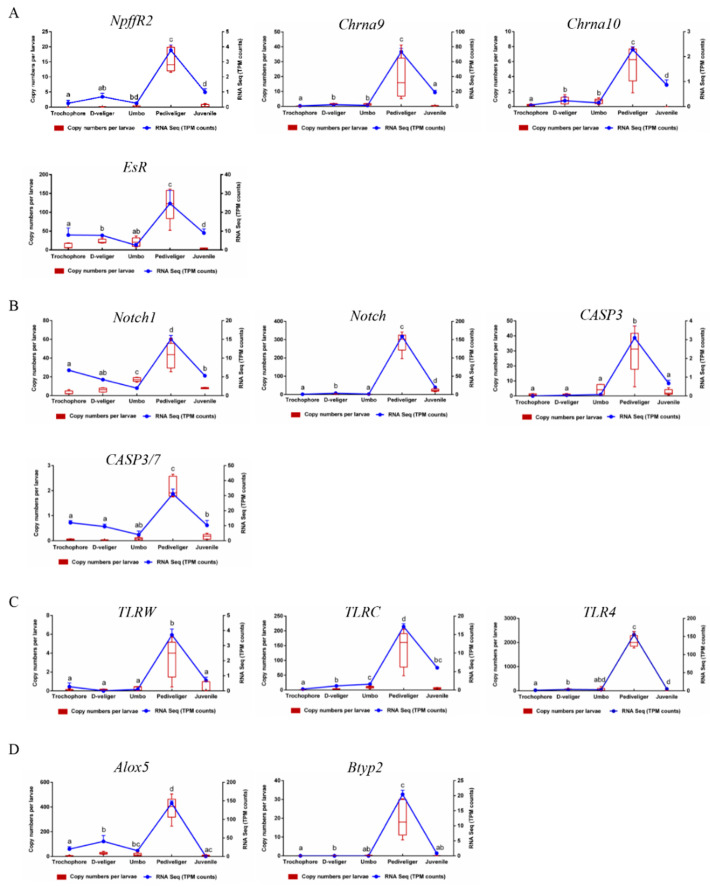
Comparison of gene expression among five developmental stages by RNA-seq and qPCR analysis. Boxplots represent the copy numbers per larvae performed by qPCR. Blue line represents the expression levels of the TPM counts assessed by RNA-seq. Analysis genes were related to neuroendocrine (**A**), growth and apoptosis (**B**), immune (**C**), and others (**D**). *NpffR2*: Neuropeptide FF receptor 2; *Chrna9*: neuronal acetylcholine receptor subunit alpha-9 isoform X1; *Chrna10*: neuronal acetylcholine receptor subunit alpha-10-like; *EsR*: estrogen receptor; *Notch1*: neurogenic locus notch homolog protein 1-like isoform X2; *Notch*: putative neurogenic locus Notch protein-like; *CASP3*: caspase-3-like 1 protein, partial; *CASP3/7*: caspase 3/7-4; *TLRW*: toll-like receptor W; *TLRC*: toll-like receptor c; *TLR4*: toll-like receptor 4 isoform X2; *Alox5*: arachidonate 5-lipoxygenase isoform X1; *Btyp2*: byssal tyrosinase-like protein 2. Different letters indicate statistically significantly different copy numbers of the qPCR analysis (*p* < 0.05).

Thirteen genes highly expressed in the pediveliger stage with >two-fold difference in expression compared to all other stages and of functional interest were selected to corroborate the outcome of the transcriptome data by qPCR (Table S1, Figure 5). All the selected genes, such as neuropeptide FF receptor 2 (*NpffR2*), estrogen receptor (*EsR*), byssal tyrosinase-like protein 2 (*Btyp2*), caspase-3-like 1 protein (*CASP3*), toll-like receptor W (*TLRW*), neurogenic locus notch homolog protein 1-like isoform X2 (*Notch1*), and arachidonate 5-lipoxygenase isoform X1 (*Alox5*), were significantly increased at the pediveliger stage relative to other stages (Figure 5, *p* < 0.05). Most of the genes revealed a similar expression pattern compared with the RNA-seq data.

## 4. Discussion

Free-swimming larvae of many marine invertebrates after fertilization ensure that larvae rapidly spread, and this may explain their rapid colonization in new habitats [30]. Larval settlement and metamorphosis are vital biological processes for survival. Larval settlement behavior starts with the sensing of external cues released, for example, from biofilms [6] that induce mussel pediveliger larvae to descend, crawl across, and attach to the appropriate substrates [7]. Morphological changes such as tissue remodeling and development occur during metamorphosis [4,31]. The genetic changes underlying the larval settlement and metamorphosis of *M. coruscus* were unveiled in the present study and provide insight into the complexity of this transformation. Based on the outcome of the DEG analysis, it is hypothesized that (1) larvae may detect and respond to the chemical cues through immune molecules, and (2) multiple neuroendocrine signaling pathways are activated and presumably involved in mussel larval metamorphosis (Figure 5). Of note was the high level of gene transcripts linked to organ development and remodeling and the importance of byssus formation during metamorphosis, since transcripts for adhesive proteins were highly represented.

### 4.1. Immune-Related Genes Involved in Larval Metamorphosis

The free-swimming larvae and juveniles of marine invertebrates are susceptible to numerous bacterial and viral pathogens [32,33]. Immune functions are generally associated with phagocytosis, encapsulation, and antimicrobial substance production to protect against pathogens [7]. In the ascidian *Boltenia villosa*, the upregulation of immune-related genes during ontogenesis is part of the programmed development of the immune system but also participates in larval settlement and metamorphosis [34]. Furthermore, the increased expression of innate immune-related genes during larval metamorphosis in *B. villosa* is also linked to the resorption and reorganization of larval tissues [34].

**Figure 6:**
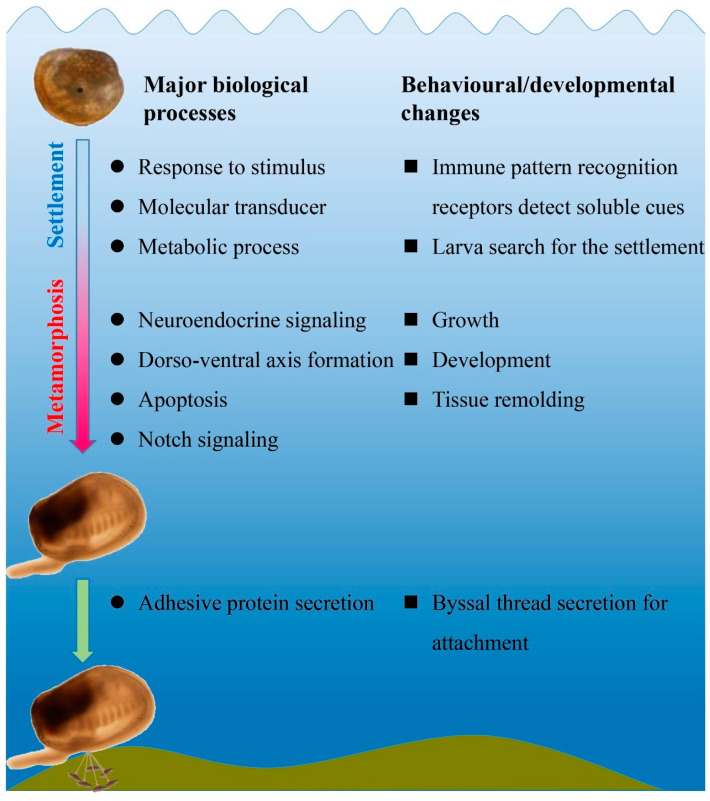
A schematic depiction of a larval settlement and metamorphosis model based on the genes identified by RNA-seq.

Biofilms and their bacteria release inductive chemical cues that mediate the larval settlement and metamorphosis of many marine invertebrates [6,8,35,36,37,38,39]. In the mussel *M. galloprovincialis*, the expression of various immune-related genes increased steadily from a trochophore larva metamorphosed to become a post-larvae when certain environmental stimulus exists, indicating the innate immunity development may be crucial for the recognition of bacterial settlement chemical cues during larval development stages [30]. In *M. coruscus*, a variety of innate immune-related genes were highly expressed during the metamorphic stage (Figure 4, Table S4) and presumably, as occurs in ascidians, are involved in the resorption and reorganization of larval tissues [34]. Identification in the pediveliger stage of immune-related DEGs involved in pathogen recognition, such as Toll-like receptors, NF-kappa B, RIG-like receptors, and NOD receptors (Figure 4, Table S3), and the role of chemical cues released from bacteria for settlement raise interesting questions. The bacterially promoted larval metamorphosis of many marine invertebrates is a widespread communication pathway that provides clues for elucidating crosstalk between metamorphosis and immune responses [7,8,30,40,41,42]. We propose that, in addition to a role in tissue remodeling, some of the immune molecules upregulated in pediveliger larvae may detect and trigger settlement and metamorphosis (Figure 6).

### 4.2. Neuroendocrine Signaling in Metamorphosis

Neurotransmitters such as epinephrine (EPI), serotonin (5-HT), acetylcholine (ACh), GABA, and L-DOPA both effectively trigger larval metamorphosis in many bivalve species [9,26,27,28,43,44,45]. The widely accepted theory has been proposed that the neuroendocrine pathways participate in larval metamorphosis in mollusks, including adrenergic, serotonergic, cholinergic, GABAnergic, and dopaminergic pathways based on various receptors activated or deactivated by the addition of receptor agonists and antagonists, respectively [7,27,29]. Molecular evidence is required to consolidate these findings, such as the selective gene knockdown methods [10,46]. In the present study, the transcriptomic analysis also revealed that some neural pathways together with their receptor genes such as *NpffR2*, *Chrna9*, and *Chrna10* were enriched at the pediveliger stage (Figure 5A, Table S4), coinciding with the neurotransmitters (EPI, 5-HT, and ACh), and promoted larval metamorphosis of the mussel *M. coruscus* [28]. The release of neurotransmitters/neuropeptides such as ACh and FMRFamide-derived neuropeptides were regulated by arachidonic acid signaling in response to the extrinsic neurotrophic factor in mollusk *Lymnaea stagnalis* [47], which is coherent with the upregulated *Alox5* (arachidonate 5-lipoxygenase isoform X1) at the pediveliger stage (Figure 5D). Furthermore, a significantly increased expression of the estrogen receptor (*EsR*) was found in pediveliger larvae compared to the other developmental stages (Figure 5A, Table S2). Although molluscan estrogen receptors and regulatory functions are still in debate [48,49], further study is needed to identify its role during metamorphosis.

Metamorphosis in vertebrates such as amphibians and teleost are driven by the thyroid hormone (THs) signaling pathway [50,51]. In both Urochordates (ascidians) and Cephalochordates (amphioxus), exogenous THs accelerate larval metamorphosis [52,53]. Studies of the TH-induced larval metamorphosis of abalone *Haliotis discus discus* and *H. gigantea* also support a role for the TH signaling pathway in the larval metamorphosis of mollusks [54]. However, although elements of the thyroid system such as thyroid peroxidase and iodothyronine deiodinase genes are present in many bivalve species such as the scallop *Chlamys farreri*, oyster *C. gigas*, and abalone *H. diversicolor* [55,56,57], their function remains largely unknown. The function of THs is mediated by the thyroid hormone receptor (TR) and retinoid X receptor (RXR) [58], and the demonstration that “competence” of pediveliger larvae for the metamorphic transition involved TR in the mussel *M. coruscus* supports a role for the thyroid system [4]. Two homologs of iodothyronine deiodinase genes were increasing expressed during ontogeny in the mussel *M. coruscus*, and anti-thyroid compound (methimazole and propylthiouracil)-affected larval metamorphosis and juvenile growth might indicate a hormonal action [24]. We further revealed that the knockdown of two iodothyronine deiodinase genes of *M. coruscus* repressed pediveliger larval metamorphic induction by epinephrine [25]. In addition, TR-interacting protein and retinoic acid receptor RXR-like genes were widely expressed during *M. coruscus* ontogenesis (Table S6), suggesting those TH signaling elements are essential for development (Figure 6). More hypotheses are still needed to be uncovered, such as whether mussels could utilize the iodide from consuming microalgae to iodinate their proteins. It remains an open question of the presence of TH signaling in many marine invertebrates. This evidence could provide clues to explore the ancient role of TH signaling during evolution.

### 4.3. Genes Involved in Development and Apoptosis

Many genes from essential pathways during larval development were annotated in *M. coruscus*, such as the Notch signaling pathway, apoptosis, dorsoventral axis formation, and axon regeneration. Notch signaling is considered an evolutionarily conserved cell interaction mechanism, which plays a fundamental role in determining cells’ fate during the development of invertebrate and vertebrate species [59,60]. Notch signaling has a significant effect on proliferation, differentiation, and apoptotic programs, influencing organ formation and morphogenesis [61]. In annelid *Platynereis dumerilii*, Notch signaling has been associated with chaetal sac formation (locomotory and mechanosensory bristles) [62], as well as the segmentation process in *Helobdella robusta* [63]. In Bryozoan *B. neritina*, Notch signaling may be involved in controlling the proliferation of fibroblasts [64]. In mollusks, the Notch signaling pathway seems to be functional in shell deposition and shell coloration associated with the calcium signaling process in the clam *Meretrix meretrix* and Pacific Oyster *C. gigas* [65,66]. Notch signaling was enriched at the pediveliger stage of *M. coruscus*, which is coherent with the significant remodeling (shell, gill, foot, and eye-spot) required to form the planktonic to benthic transition in pediveliger larvae. For example, a transcription factor 4 (*HES4*) gene was significantly upregulated in pediveliger larvae (Table S4).

Apoptosis is an essentially biological process during animal development, which is used by the animal to ablate their tissue architecture [67]. Apoptosis is involved in the metamorphosis of marine invertebrates [68,69], and apoptotic genes such as caspases regulate the loss of the tail in the ascidian *Ciona intestinalis* [70]. In the purple sea urchin *Strongylocentrotus purpuratus*, gene knockdown experiments have shown that apoptosis is essential for the metamorphic transition [71]. Similarly, in the bivalve oyster (*C. angulate*), two caspase genes are important in tissue regression during larval metamorphosis [70]. The role of the apoptosis pathway in the metamorphosis of *M. coruscus* is clear, and 13 apoptosis-related genes (10 upregulated and 3 downregulated genes) were identified as differentially expressed in the pediveliger larvae (Figure 4, Tables S4 and S5). Of note was the identification of upregulated caspase genes corroborating the role of caspases in bivalve tissue remodeling during metamorphosis. However, the transcriptome data provided further insight and other important apoptosis-related cues during metamorphosis, which deserve further investigation.

### 4.4. Adhesive Protein Genes in Metamorphosis

Some bivalve species, such as mussels and oysters, secrete adhesives to allow them to be temporarily or permanently attached to the substrate in the underwater environment [72]. Mussel byssus, secreted by their foot, is an extracellular proteinaceous fiber composed of a variety of mussel foot proteins [73,74]. A mussel foot has sensory, locomotory, and secretory roles that help mussels in underwater adhesion [75]. Collagens, matrix proteins, and polyphenolic proteins were the main components of a byssal thread [72]. Our data revealed that collagen protein genes were abundantly expressed at the pediveliger stage compared to the other stages (Table S2). This might indicate that the pediveliger larvae synthesize the structural proteins for secreting byssal threads prior to metamorphosis [76]. Furthermore, many sequences containing a von Willebrand Factor type A (vWF-A) domain-containing protein were highly enriched at the pediveliger stage (Table S2). The vWF-A domain genes have been reported to bind with collagen, which might involve the adhesive structure formation by the regulation of protein aggregation [76,77]. Additionally, some byssal-related enzyme genes, including byssal tyrosinase-like protein 2 (*Btyp2*), byssal metalloproteinase inhibitor-like protein 1, byssal glycosyl-hydrolase-like protein 2, and byssal peroxidase-like protein 1, were highly expressed at the pediveliger stage (Figure 5D, Table S2). Those byssal-related enzymes such as peroxidases and tyrosinases are the essential enzymes mediating the formation of dityrosine bonds, DOPA, and DOPA–quinone groups involved in the biosynthetic pathway of the mussel byssus [72,76].

## 5. Conclusions

Our transcriptome study identified differentially expressed genes at the pediveliger stage in *M. coruscus*. Gene expression profiles associated with metamorphic morphogenesis in larval organ development and remodeling were revealed by the enrichment of apoptosis, dorsoventral axis formation, axon regeneration, and Notch signaling pathways at the pediveliger stage. Furthermore, the enrichment of genes of the immune and neuroendocrine pathways supports a role for these systems in mussel metamorphosis. The data obtained provide the basis for future studies to disentangle gene networks and the biology of larval settlement and metamorphosis.

## Data Availability

The raw sequence data of the developmental stages have been submitted to GenBank (accession number: SRP300586). Further inquiries can be directed to the corresponding author.

## Supplementary Materials

The following supporting information can be downloaded at: www.mdpi.com/article/10.3390/genes14020287/s1: Table S1: The primer pairs used in qPCR are presented together with the amplicon size, melting temperature, r2, and efficiency. NpffR2: Neuropeptide FF receptor 2; Chrna9: neuronal acetylcholine receptor subunit alpha-9 isoform X1; Chrna10: neuronal acetylcholine receptor subunit alpha-10-like; EsR: estrogen receptor; Notch1: neurogenic locus notch homolog protein 1-like isoform X2; Notch: putative neurogenic locus Notch protein-like; CASP3: caspase-3-like 1 protein, partial; CASP3/7: caspase 3/7-4; TLRW: toll-like receptor W; TLRC: toll-like receptor c; TLR4: toll-like receptor 4 isoform X2; Alox5: arachidonate 5-lipoxygenase isoform X1; Btyp2: byssal tyrosinase-like protein 2; Table S2: Differentially expressed genes (DEGs) with increase (|log2 FC| ≥ 1) in the pediveliger stage compared to all other stages. Gene id, annotation, and average TPM counts/stage (n = 4) is indicated; Table S3: Differentially expressed genes (DEGs) with decrease (|log2 FC| ≥ 1) in the pediveliger stage compared to all other stages. Gene id, annotation, and average TPM counts/stage (n = 4) is indicated; Table S4: KEGG pathway classifications of the DEGs with increase (|log2 FC| ≥ 1) in the pediveliger stage compared to all other developmental stages; Table S5: KEGG pathway classifications of the DEGs with decrease (|log2 FC| ≥ 1) in the pediveliger stage compared to all other developmental stages; Table S6: BLAST results against the GO/KEGG/COG/NR/Swiss-Prot/Pfam databases for all the distinct sequences with a cut-off E value above 10^-5^ are shown. Gene id, annotation, and average TPM counts/stage (n = 4) is indicated.
